# Bisphenol A Disrupts Ribosome Function during Ovarian Development of Mice

**DOI:** 10.3390/toxics12090627

**Published:** 2024-08-26

**Authors:** Xin Ma, Yongjie Wang, Weiqi Li, Kaiyue Wang, Shilei Zhang

**Affiliations:** 1 College of Animal Science and Technology, Shihezi University, Shihezi 832003, China; mx1579356366@163.com (X.M.); 18888572758@163.com (W.L.); 13904595077@163.com (K.W.); 2 Department of Animal Sciences, College of Agriculture and Environmental Sciences, North Carolina Agricultural and Technical State University, Greensboro, NC 27411, USA; ywang3@ncat.edu; 3 Xinjiang Tycoon Group Co., Ltd., Changji 831100, China

**Keywords:** Bisphenol A, ovarian development, apoptosis, transcriptome, ribosome

## Abstract

This study examines the impact of Bisphenol A (BPA), a prevalent environmental estrogenic toxicant, on the ovarian development of mice. Mice were exposed to varying BPA doses from in utero to postnatal stages, up to weaning (day 21, PND 21) and puberty (day 45, PND 45). The BPA content in the serum of the offspring mice on PND 45 was higher than that of the mice sacrificed at PND 21. However, the ovary organ index of the mice of PND 21 was significantly increased, and the ovarian structure was damaged when exposed to BPA. In contrast, the mice with PND 45 did not show apparent ovarian lesions. On the other hand, granulosa cell apoptosis was detected in both PND 21 and PND 45 mice ovaries, and *ERβ* was increased under the influence of BPA. Transcriptomic analysis revealed BPA’s significant impact on ribosomal gene expression, marked downregulation of *Rpl21* and *Rpsa*, and upregulation of *Rps2* in both age groups. These transcriptomic alterations were further corroborated by real-time PCR, highlighting a dose-dependent effect of BPA on *Rps2*. Our findings confirm BPA’s detrimental effects on ovarian health, with more pronounced damage in younger mice, suggesting heightened vulnerability in this group. The study underscores ribosomes as critical targets in BPA-induced ovarian developmental disruptions.

## 1. Introduction

Bisphenol A (BPA) is a crucial component in manufacturing synthetic plastics and is also found in certain waterproof and anti-corrosive coatings. Its widespread presence in the environment poses potential risks to animals’ and humans’ reproductive and developmental health [1]. BPA is considered to have estrogen activity, and studies have shown that a lower concentration of BPA can affect aquatic organisms’ reproduction [2,3,4]. BPA pollution is most severe in Asia, where the highest sediment concentrations of BPA surpass those found in Europe [5]. BPA has been found in plastic-barreled white wine [6] and has also been detected in canned beer [7]. In addition, BPA has been detected in human urine, serum, and other liquids, and the concentration may even be higher than the dose used in in vitro culture studies [8,9]. In a study in North America, high levels of BPA were detected in maternal serum [10]. BPA can cross the placental barrier, allowing transmission to the fetus. Additionally, its presence can be seen in both fetal and neonatal serum. [11]. Exposure to low doses of BPA has been demonstrated to interfere with multiple signaling pathways, disrupting their normal functions [12]. Investigations into BPA’s interactions with various pathways, including NF-κB, JNK, MAPK, ER, and AR, have revealed its potential role in altering cellular morphology and potentially contributing to the development of tumors [13].

Studies have shown that BPA can bind to the estrogen receptor (ER), and its affinity for *ERβ* is about 10 times higher than that of *ERα* [14]. Previous studies have shown that BPA can stimulate multiple pathways in cells at a low concentration. The U.S. Department of Health and Human Services National Toxicology Program–Center for the Evaluation of Risks to Human Reproduction (NTP-CERHR) indicates that BPA > 50 mg/kg causes abnormal puberty development [15]. However, the impact of BPA on the body is complex, and very low doses can have a significant effect [16]. The low-dose effects of BPA include prostate cancer, breast cancer, uterine lesions, estrous cycle disorders, precocious puberty, and genital malformations [17,18]. BPA can cause endocrine disorders, leading to ovarian dysfunction, inducing apoptosis of ovarian granulosa cells, and leading to ovarian DNA damage [19,20]. Exposure to BPA in mothers can impact the endocrine health of their offspring, and BPA exposure during the perinatal period has been linked to ovarian dysfunction in the next generation [21].

Consequently, investigating the detrimental impacts of prolonged, low-dose BPA exposure on female reproductive functions holds substantial importance. This study examines the effects of oral BPA exposure, ranging from 0.05 mg/kg to 50 mg/kg body weight per day, on ovarian development in F1 female mice.

## 2. Materials and Methods

### 2.1. Animals and Chemicals

One hundred male and 200 female Kunming mice, all eight weeks old, were acquired from SPF Biotechnology Co., Ltd. (Beijing, China). Certified as clean grade and Specific Pathogen Free (license number: SCXK (Beijing) 2016-0002), these mice underwent a week-long acclimatization period. For breeding, one male and two females were housed together overnight, with the detection of a vaginal plug in the morning, marking gestation day (GD) 0. The pregnant mice were randomly allocated into seven groups, each consisting of 20. Group A served as the control with 0.00 mg/kg (BPA/Bodyweight) BPA exposure. Groups B, C, D, E, F, and G were subjected to varying BPA dosages: 0.05, 0.5, 5, 10, 20, and 50 mg/kg (BPA/Bodyweight) per day, respectively. All mice were fed in metal cages with glass drinking bottles. Water intake was monitored to ensure all the mice received similar dosing. BPA exposure, administered via drinking water, spanned from GD 0 to the end of lactation. Post-weaning at postnatal day (PND) 21, select female offspring with similar body weight from different litters were euthanized, while others continued BPA exposure until PND 45. The total exposure period for offspring females ranged from 39 to 63 days, including fetal development. The reproductive capabilities of F1 and F2, such as average fetus number, fetus weight, death rate, and female/male rate, were measured, and the data are shown in Supplementary Tables S1 and S2. The water intake of F1 during the pregnancy and lactation was measured and shown in Supplementary Tables S3 and S4. All procedures adhered to NIH guidelines for laboratory animal care and received approval from the Animal Welfare Committee of Shihezi University, China (No. IACECHSHZU20221017). BPA (≥99% purity, Sigma, Livonia, MI, USA) was prepared as a 50 mg/mL stock solution in distilled water with 0.5 mol/L NaOH and 25% ethanol, stored at 4 °C. This solution was then diluted to the required concentrations as needed. The control group was only provided with water containing 0.5 mol/L NaOH and 25% ethanol. The final concentration of NaOH in each group was 0.5 mmol/L, and the ethanol was 0.025%.

### 2.2. Sample Collection

On postnatal days (PND) 21 and 45, 20 female F1 mice with similar body weights in each group (from at least 6 different litters) were humanely euthanized under anesthesia when they were in the diestrus. Euthanasia of the mice was carried out using carbon dioxide (CO_2_) inhalation, following the guidelines recommended by the Institutional Animal Care and Ethics Committee of Shihezi University. The mice were exposed to a gradually increasing concentration of CO_2_ in a controlled chamber to ensure a humane, quick, and painless death. The CO_2_ displacement rate was maintained at 20–30% of the chamber volume per minute, consistent with the standard ethical practices to minimize animal distress. Following CO_2_ administration, the absence of vital signs was confirmed in each mouse to ensure humane and effective euthanasia. Blood was collected from the tail of each mouse before euthanasia, and serum samples were extracted and preserved at −80 °C. The ovaries were then harvested and weighed, with the ovary index calculated as the ratio of the combined ovarian weight (in mg) to body weight (in g). Ovary samples were preserved at −80 °C for RNA extraction. For histological analysis, ovarian tissues were fixed in 4% paraformaldehyde, embedded in paraffin, and sectioned into 5 µm slices.

### 2.3. Total BPA in Serum Determination

Total BPA levels in the serum were quantified using the Bisphenol A test kit used for mouse blood with a detection range from 10 to 10,000 ng/L (Shanghai Runyu Biotechnology Co., Ltd., Shanghai, China, Lot No: RY-12919), following the manufacturer’s guidelines. It measured both free and conjugated BPA, and there was no cross-reactivity with other bisphenols.

### 2.4. Immunohistochemistry Staining

The ovarian tissue sections obtained from the samples were subjected to immunohistochemical staining to evaluate changes in the expression levels of *ERβ*. This involved using primary antibodies–Mouse monoclonal *ERβ* antibody (ab187291, Abcam, Waltham, MA, USA). The secondary antibody applied was an HRP Conjugated Mouse IgG antibody (SV0001, Boster Biotech Co., Ltd., Wuhan, China). The paraffin sections were first deparaffinized in xylene, then rehydrated in a graded alcohol series (100% alcohol, 95% alcohol, 90% alcohol, 80% alcohol, 70% alcohol, and ddH2O, respectively). After rehydration, heat-induced antigen retrieval was performed using sodium citrate buffer (10 mM sodium citrate, 0.05% Tween 20, pH 6.0), followed by quenching in 3% H_2_O_2_ for 10 min to eliminate endogenous peroxidase. After incubation, the samples were washed with phosphate-buffered saline (PBS) and then blocked with 5% BSA (Thermo Scientific, Waltham, MA, USA) for 5 min. This was followed by incubation with primary antibodies at 37 °C for 3 h. After washing 3 times with PBS, they were incubated with secondary antibodies at 37 °C for 30 min, washed with PBS, and then developed with 3,3′-diaminobenzidine (DAB) for 3 min, where brown-yellow indicates the expression of the corresponding protein.

### 2.5. Apoptosis of Ovarian Cells

The apoptosis of each ovary sample was examined using the TUNEL kit (TUNEL Apoptosis Detection Kit, 11684817910, Roche, Indianapolis, IN, USA). The images of the slides were taken using a microscopic imaging system (Olympus BX43, Bartlett, TN, USA). The quantification of the apoptosis of each sample was determined by the optical density via Image J [22]. To quantify apoptosis in each sample using Image J, we first opened the stained tissue images in the software and converted them to 8-bit grayscale to standardize the analysis. Regions of interest (ROIs) representing areas with apoptosis were selected using the “Polygon” or “Freehand” tool. The optical density (OD) within each ROI was measured via Analyze > Measure, with background noise subtracted by measuring OD in non-stained areas. The resulting OD values were then exported for statistical comparison, allowing for a quantitative assessment of apoptosis across samples.

### 2.6. RNA Extraction and Quality Determination

Ten ovaries were randomly chosen from each experimental group to extract total RNA, which was then used for both transcriptome sequencing and real-time PCR analyses. The total RNA from each ovarian sample was extracted using the Eastep Super Total RNA Extraction Kit (LS1040, Promega, Madison, WI, USA). RNA degradation and potential contamination were evaluated via agarose gel electrophoresis (712BR, Bio-rad, Hercules, CA, USA). At the same time, RNA purity and concentration were determined using Nanodrop (Q33226, Thermo Scientific, Waltham, MA, USA) (OD260/280 ratio) and Qubit (Q33240, Invitrogen, Waltham, MA, USA), respectively. Agilent 2100 (G2939A, Agilent, Santa Clara, CA, USA) was used to assess RNA integrity accurately. Following quality assessment, eukaryotic mRNA is enriched with magnetic beads (80101G, Invitrogen, Waltham, MA, USA) bound to Oligo (dT), fragmented, and reverse-transcribed into cDNA using random hexamers. This cDNA was then purified, end-repaired, A-tailed, adaptor-ligated, size-selected, and PCR-enriched to create the final cDNA library. The library underwent initial quantification with Qubit 2.0, insert size verification with Agilent 2100, and precise concentration measurement using qPCR to ensure a quality library with an adequate concentration of >2 nM.

### 2.7. Total RNA Sequencing

Transcriptome sequencing was performed on ovarian samples from both the control and 50 mg/kg BPA-exposed groups, utilizing the Illumina HiSeq sequencing platform. The RNA-seq reads were pre-processed using a custom Perl script based on a filtration algorithm to remove unreliable reads and trim low-quality sections [23]. The FPKM (Fragments Per Kilobase of transcript per Million mapped reads) values of sequenced genes were counted with the method according to [24]. Differential expression analysis was performed using EBSeq package [25]. Genes with log2(fold change) ≥ 1 and false discovery rate (FDR) < 0.01 were considered significant for differentially expressed genes (DEGs). Post-sequencing, the differentially expressed genes (DEGs) were annotated using the Gene Ontology (GO) (https://geneontology.org/, accessed on 1 December 2023) and Kyoto Encyclopedia of Genes and Genomes (KEGG) (https://www.genome.jp/kegg/, accessed on 1 December 2023) databases for comprehensive analysis. These DEGs were then further validated through real-time PCR.

### 2.8. Real-Time PCR

For cDNA synthesis, the Advantage RT-for-PCR Kit (Code No. 639505, TAKARA, Shiga, Japan) was utilized. The reverse transcription protocol involved a two-step process: incubation at 42 °C for 15 min followed by 85 °C for 15 s. The PCR reaction mix, with a total volume of 10 μL, consisted of 5 μL Roche FastStart Universal SYBR Green Master, 3 μL ddH_2_O, 0.75 μL of forward primer, 0.75 μL of reverse primer, and 0.5 μL of cDNA sample (concentration 5 ng/μL). Primer concentrations were maintained at 0.1 μmol/mL, and details of the primers are provided in Table 1. The length of the amplified fragment was a key consideration in the design of each gene’s primer. The PCR conditions were set as follows: an initial denaturation at 95 °C for 10 min, followed by 40 cycles of 95 °C for 30 s and 60 °C for 30 s. Melting curve analysis was conducted at 95 °C for 10 s and 65 °C for 60 s, with a gradual increase to 95 °C and then a decrease to 37 °C at a rate of 0.2 °C/s.

### 2.9. Statistical Analysis

Immunohistochemistry and TUNEL assay results were quantitatively analyzed using Image J software. The gathered data were then inputted into SPSS 19.0 for statistical analysis. Results are presented as mean ± standard error ($\bar{x}$ ± SEM). Statistical evaluations were performed using one-way ANOVA and the Chi-square test (χ²), with comparisons made against the control group. A *p*-value of less than 0.05 was considered statistically significant, denoted as (*). The reliability of the measurement results was assessed by calculating the linear regression coefficient R² and the regression equation from the standard curve provided in the kit. An R² value of 0.92 or higher indicates dependable results. In our analysis, an R² of 0.9977 was obtained, confirming the reliability of the measurements.

## 3. Results

### 3.1. BPA Exposure Increased BPA Contents in Serum F1 Females

**Figure 1:**
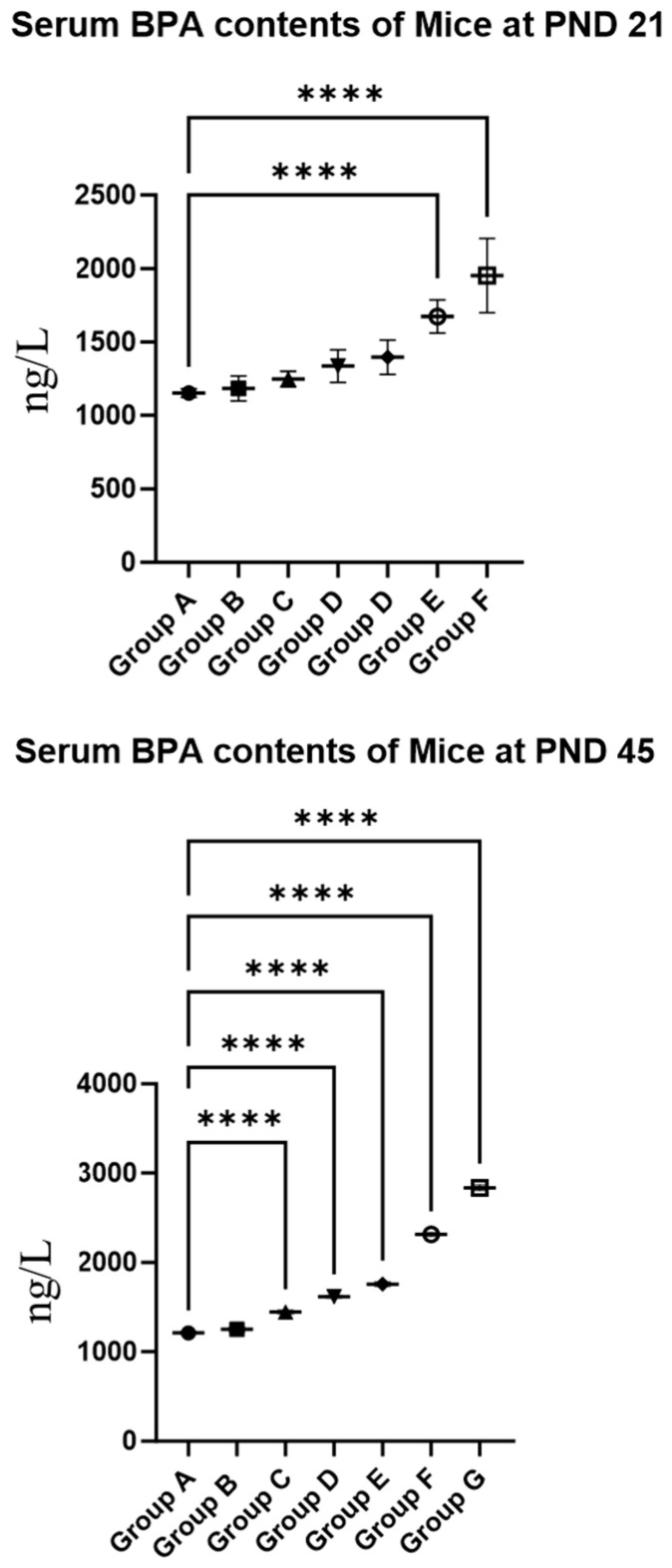
Serum BPA contents in offspring females. Note: Group A served as the control group. Group B received 0.05 mg/kg BPA, Group C 0.5 mg/kg, Group D 5 mg/kg, Group E 10 mg/kg, Group F 20 mg/kg, and Group G 50 mg/kg BPA. Twenty mice for each group. “****” indicates significant differences between the same dose group of different ages (*p* < 0.0001).

As presented in Figure 1, the data revealed that for 21-day-old female mice exposed to BPA doses of 20 mg/kg or higher, serum BPA levels were significantly elevated compared to the control group (*p* < 0.05). In 45-day-old females, a BPA dose of 0.5 mg/kg or higher resulted in significantly higher serum BPA levels than those in the control group (*p* < 0.05). A comparison between the two age groups showed that at a BPA dose exceeding 10 mg/kg/day, serum BPA levels in PND 45 mice were significantly higher than in PND 21 mice (*p* < 0.05). These findings suggest that BPA exposure increases serum BPA concentrations in mice.

### 3.2. BPA Exposure Elevates the Body Weight, Ovarian Weight, and Ovarian Organ Index of 21-Day-Old F1 Females

**Figure 2:**
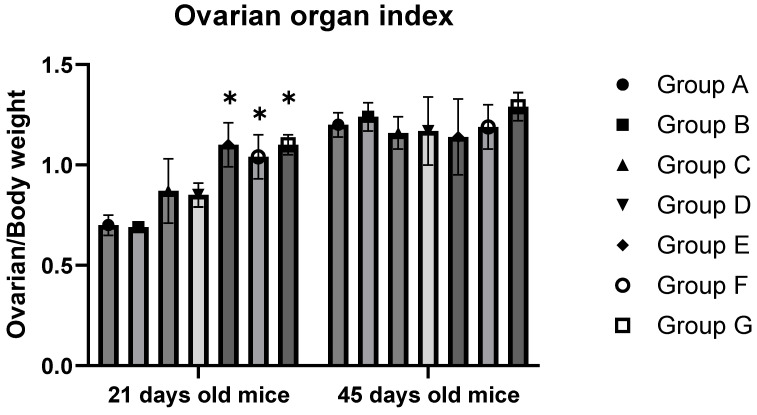
Ovarian organ index of mice. Note: Group A served as the control group. Group B received 0.05 mg/kg BPA, Group C 0.5 mg/kg, Group D 5 mg/kg, Group E 10 mg/kg, Group F 20 mg/kg, and Group G 50 mg/kg BPA. Twenty mice for each group. “*” indicates significant differences between the same dose group of different ages (*p* < 0.05).

Table 2 and Figure 2 display the data related to the mice’s body weight, ovarian weight, and ovarian organ index. The findings indicate that for 21-day-old F1 mice, a significant increase in ovarian organ weight and index was observed at a BPA exposure level of 10 mg/kg (*p* < 0.05). In contrast, the mice’s bodies decreased significantly with increased BPA concentration (*p* < 0.05). In the groups exposed to 10, 20, and 50 mg/kg BPA, the ovarian organ index, also known as organ coefficient, was significantly higher than the control group (*p* < 0.05). However, in the case of 45-day-old offspring, no significant change in the ovarian index was noted across varying levels of BPA exposure (*p* > 0.05).

### 3.3. Apoptosis in Ovarian Granulosa Cells

**Figure 3:**
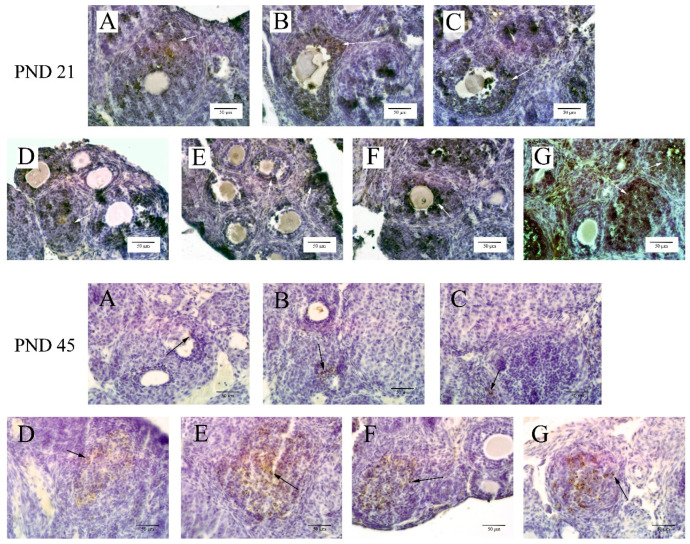
The TUNEL staining results of ovarian cell apoptosis (400×). Note: The arrow indicates apoptotic cells, mainly in ovarian granulosa cells. The scale length was 50 μm. Group **A** served as the control group. Group **B** received 0.05 mg/kg BPA, Group **C** 0.5 mg/kg, Group **D** 5 mg/kg, Group **E** 10 mg/kg, Group **F** 20 mg/kg, and Group **G** 50 mg/kg BPA. Twenty mice for each group.

Utilizing the TUNEL kit for staining the sample slices revealed that ovarian cell apoptosis predominantly occurred in granulosa cells, as indicated by arrows in Figure 3. The optical density values for these cells are detailed in Table 3. The results demonstrated that at 20 mg/kg or higher BPA concentrations, the apoptosis rate in ovarian granulosa cells of 21-day-old offspring was significantly greater than in the control group (*p* < 0.05). In contrast, for 45-day-old offspring mice, significant induction of apoptosis in ovarian granulosa cells was observed only at the highest BPA exposure level of 50 mg/kg.

### 3.4. ERβ Expressions in the Ovary of F1 Females

**Figure 4:**
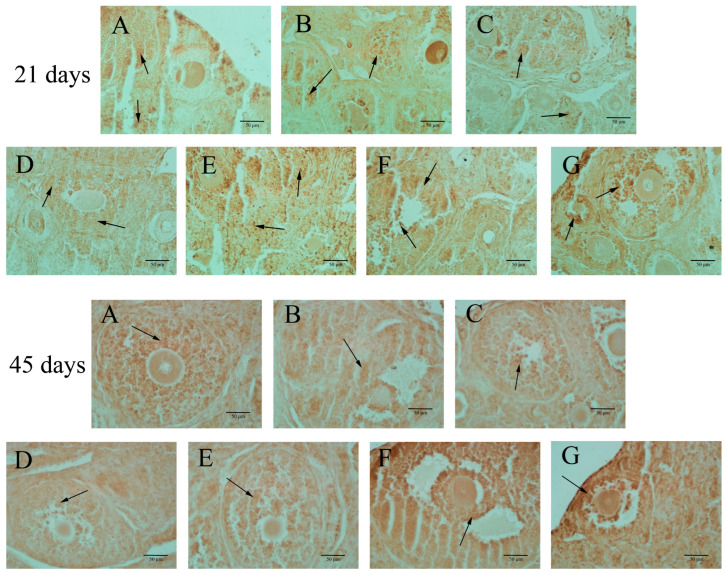
Ovarian *ERβ* expression (400×). Note: The arrow indicates apoptotic cells, mainly in ovarian granulosa cells. The scale length was 50 μm. Group **A** served as the control group. Group **B** received 0.05 mg/kg BPA, Group **C** 0.5 mg/kg, Group **D** 5 mg/kg, Group **E** 10 mg/kg, Group **F** 20 mg/kg, and Group **G** 50 mg/kg BPA. Twenty mice for each group.

*ERβ* expression was predominantly observed in the granulosa cells of growing follicles, as illustrated in Figure 4 and detailed in Table 4. In 21-day-old mice, BPA exposure at doses below 50 mg/kg did not affect *ERβ* expression in the ovaries. However, in 45-day-old offspring mice, BPA exposure at levels of 20 mg/kg or higher led to a significant increase in *ERβ* expression in the ovaries, as indicated in Table 4 (*p* < 0.05).

### 3.5. Differentially Expressed Genes in the Ovary

**Figure 5:**
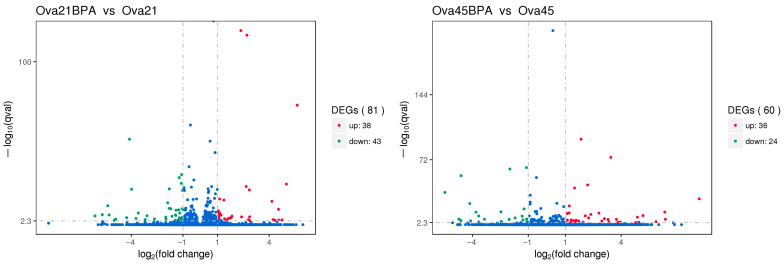
Volcano map of differentially expressed genes. Note: In the graphical representation of the gene expression data, genes with significant differential expression are marked by red dots for up-regulation and green dots for down-regulation. Genes that do not exhibit significant differential expression are shown as blue dots. The abscissa (horizontal axis) on the graph denotes the fold change of genes across different samples. In contrast, the ordinate (vertical axis) represents the negative logarithm of the statistical significance of the differential expression levels of these genes.

Transcriptome sequencing was employed to compare the RNA from the ovaries of Group G (50 mg/kg BPA group) with Group A (control group), aiming to ascertain the significance of the sequencing outcomes. The analysis revealed that, compared to the control, the ovaries of 21-day-old mice in the 50 mg/kg BPA group exhibited 81 significant DEGs: 38 were up-regulated, and 43 were down-regulated. In the case of 45-day-old offspring, 60 significant DEGs were identified, with 36 up-regulated and 24 down-regulated. These findings are presented in Figure 5.

### 3.6. Key Metabolic Pathways Affected by BPA in Ovarian Development

**Figure 6:**
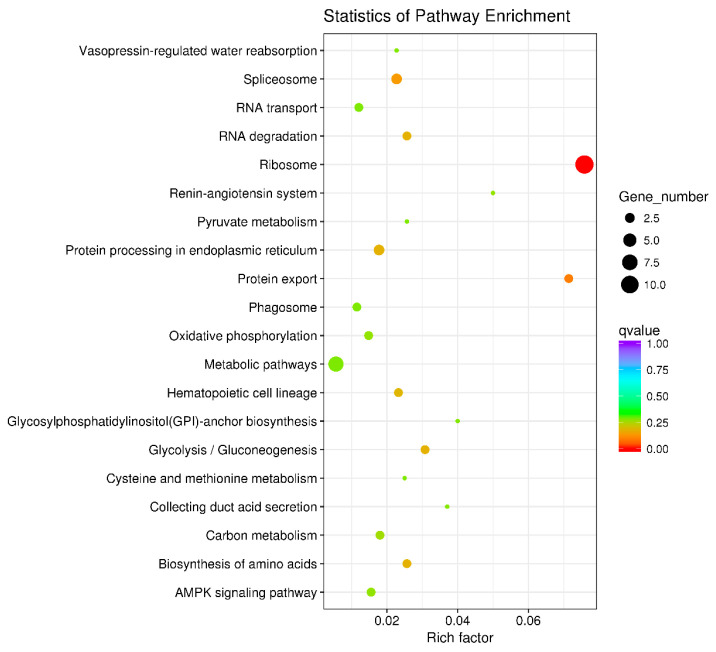
Differential gene KEGG enrichment of 21 days ovary. Note: In the graphical representation of the metabolic pathway analysis, the vertical axis denotes the names of the metabolic pathways, while the horizontal axis represents the degree of enrichment of these pathways. The size of each dot in the graph reflects the number of differentially expressed genes within a particular pathway. Additionally, the color of the dots corresponds to different Q-values, providing a visual indicator of the statistical significance of the enrichment in each pathway.

**Figure 7:**
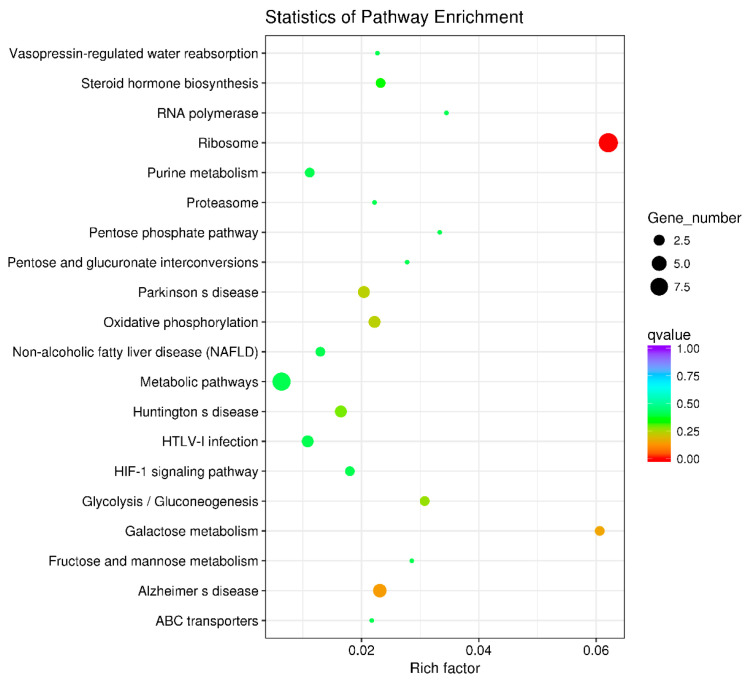
Differential gene KEGG enrichment of ovary of 45 days females. Note: In the graphical representation of the metabolic pathway analysis, the vertical axis denotes the names of the metabolic pathways, while the horizontal axis represents the degree of enrichment of these pathways. The size of each dot in the graph reflects the number of differentially expressed genes within a particular pathway. Additionally, the color of the dots corresponds to different Q-values, providing a visual indicator of the statistical significance of the enrichment in each pathway.

**Figure 8:**
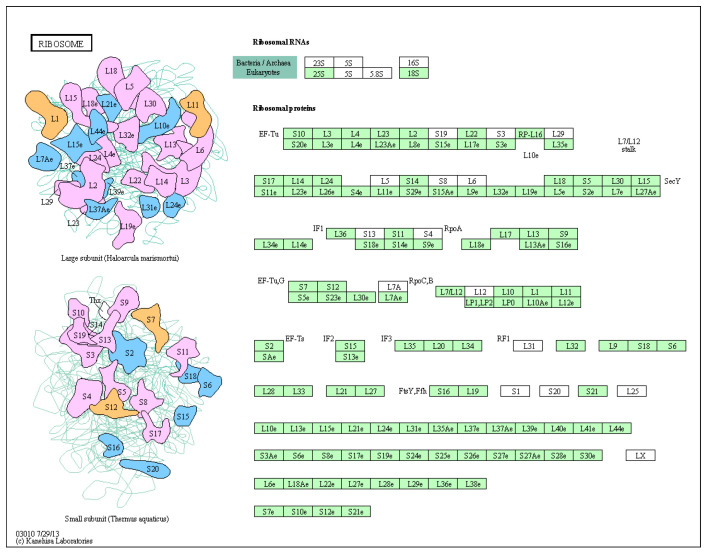
Ribosome metabolic pathway map.

KEGG enrichment analysis was utilized to examine the functional clusters of differentially expressed genes in the ovaries. The results highlighted significant enrichment in ribosomal metabolic pathways for both 21-day-old and 45-day-old mice, as depicted in Figure 6 and Figure 7. Figure 8 illustrates the specific ribosomal metabolic pathway involved. In the ovaries of 21-day-old mice, there was a notable down-regulation in the expression of ribosomal proteins L3, L21, and Sa. Conversely, the expression of ribosomal proteins S2, S28, L26, L32, and L10a was significantly up-regulated. In the ovaries of 45-day-old mice, the expression genes of ribosomal proteins L21 and Sa showed significant down-regulation, while those of L3, S2, L7a, S26, and L26 exhibited notable up-regulation.

### 3.7. Gene Expression of Rps2, Rpl21, and Rpsa

**Figure 9:**
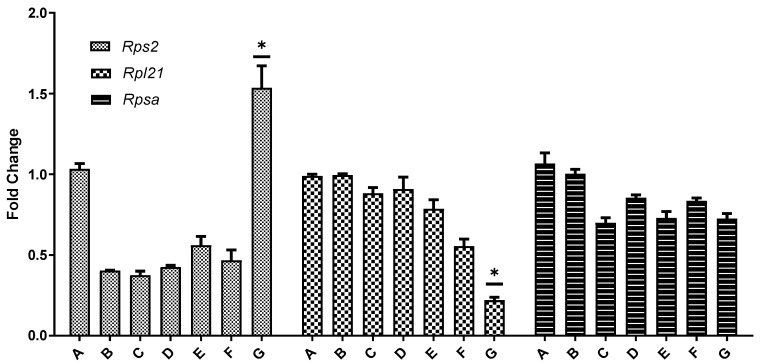

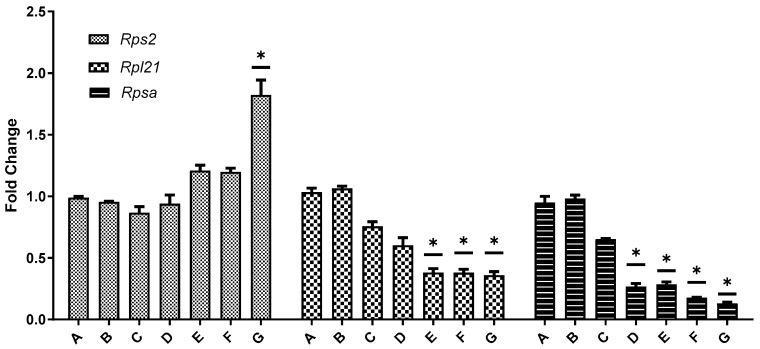
Fluorescence quantitative PCR verification of differentially expressed genes in the ovarian ribosomal pathway. Note: In the graphical representation of gene expression data, the ordinate (vertical axis) represents the fold difference in expression relative to the β-actin reference gene compared to the blank control. The abscissa (horizontal axis) denotes the seven experimental groups, categorized according to their respective BPA exposure doses. Group A serves as the control group, Group B is the 0.05 mg/kg BPA group, Group C the 0.5 mg/kg group, Group D the 5 mg/kg group, Group E the 10 mg/kg group, Group F the 20 mg/kg group, and Group G the 50 mg/kg BPA group. Twenty mice for each group. “*” indicates significant differences between the same dose group of different ages (*p* < 0.05).

Real-time PCR was employed to measure the expression levels of *Rps2*, *Rpl21*, and *Rpsa* in the ovaries across all seven experimental groups, with β-actin as the internal reference gene to correct any variance in RNA yield among different tissues. The results demonstrated a dose-dependent effect of BPA on these genes. In 21-day-old mice, the expression levels of *Rpl21* and *Rpsa* in the ovaries decreased with increasing BPA doses, with the reduction in *Rpl21* being more pronounced than *Rpsa*. This trend aligned with the transcriptome sequencing outcomes. The expression of *Rps2* varied depending on the BPA dose: it was lower than the control group at doses ranging from 0.05 mg/kg to 20 mg/kg but showed higher expression at the 50 mg/kg BPA dose. In the ovaries of 45-day-old mice, both *Rpl21* and *Rpsa* expressions decreased with increasing BPA doses, while *Rps2* expression exhibited an increase with higher BPA doses, as depicted in Figure 9.

## 4. Discussion

BPA is an essential raw material for synthetic plastics. Although BPA is unstable and has a half-life of only 1 to 5 days, its ongoing and growing use in the environment is concerning. This is because BPA remains stable for several years when it is in polycarbonate [26,27,28]. Studies have shown that BPA content in landfill leachate is up to 4500 μg/L [29]. The cashiers had a significantly higher BPA content in their blood than ordinary people due to the use of thermal paper [30]. This research focused on assessing the impact of prolonged, low-dose BPA exposure on ovarian development. The findings revealed that serum BPA levels in PND 21 mice were significantly increased at 20 mg/kg or higher BPA concentrations. In PND 45 mice, serum BPA levels significantly increased at 0.5 mg/kg or higher BPA exposures. Continuous BPA exposure markedly elevated the serum BPA levels in offspring mice, with PND 45 mice displaying significantly higher serum BPA concentrations than PND 21 mice. This observed elevation in serum BPA levels could be attributed to indirect exposure during fetal and lactation periods. PND 45 mice experienced direct BPA exposure through their drinking water from PND 21 to PND 45, in addition to the in-utero and lactation exposure, potentially contributing to the higher BPA levels observed at this stage. From the point of view of BPA in serum, it seems that prepubertal (45-day-old) mice are more vulnerable to BPA; however, considering that exposure to BPA in young children can lead to more severe consequences, it is difficult to judge who is affected more seriously [31]. However, the differences between day 21 and day 45 animals may be due to developmental stage and exposure time differences at those ages.

Low-dose (50 mg/kg body weight) exposure to BPA could affect normal physiological activities of the body [32]. In in vitro studies, it has been shown that BPA interferes with the development and maturation of eggs in vitro and inhibits granulosa cell proliferation [33]. Serum test results in patients with polycystic ovary syndrome (PCOS) have shown that BPA levels in PCOS patients are significantly higher than in healthy women, suggesting that BPA is closely related to ovarian disease and dysfunction [34]. The effect of BPA on ovarian development was confirmed by indirect exposure to higher doses (up to 1000 mg/kg/d) but with a shorter duration of exposure [35]. They exposed pregnant mice (F0) to the injection of BPA at doses of 0–1000 mg/kg BW on days 12–16 of gestation. Increased body weight and decreased relative weights of the ovary and uterus in F2 female mice from foster-bred F1 mice. Observations included the expansion or enhancement of the uterine lumen, partial loss of the uterine epithelium, and demethylation of the *HOXA10* gene in the uterus. The ovarian index of female mice in PND 21 and 45 was counted in our study. The study found that a 10 mg/kg BPA dose significantly increased the ovarian organ index in 21-day-old mice. In contrast, BPA doses up to 50 mg/kg did not significantly alter the ovary index in 45-day-old mice. Despite lower serum BPA levels in PND 21 mice compared to PND 45 mice, the ovarian damage was more severe in the younger group. This could be due to a higher resistance to BPA in PND 45 mice. While no significant pathological changes were noted in the ovaries of 45-day-old mice, it does not exclude the possibility of BPA-induced ovarian damage at this age. TUNEL assays conducted on ovarian tissues from both age groups confirmed the presence of apoptosis of ovaries, indicating that BPA exposure affected ovarian cells in both 21-day and 45-day-old mice.

BPA has weak estrogen activity and will compete with E_2_ for binding to *ERβ* [36]. ERβ regulates ovarian function, particularly in the granulosa cells, stroma, and oocytes. In granulosa cells, ERβ modulates follicular development and estrogen production, influencing cell proliferation and differentiation [37]. In the ovarian stroma, ERβ activation contributes to tissue remodeling and the regulation of local hormone production [38]. In oocytes, ERβ is involved in the maturation process, supporting the oocyte’s developmental competence and overall fertility [39]. These roles highlight the importance of ERβ in maintaining ovarian health and function at various life stages. Studies have shown that BPA can induce meiotic arrest [40]. However, previous studies have suggested that BPA can promote cell proliferation through estrogen receptors [41]. BPA has the effect of receptor inhibitors and has specific estrogen activity [42]. Our research confirmed *ER-beta* as one of the crucial receptors affected by BPA. Immunohistochemistry results of the current study showed that exposure to BPA caused changes in *ERβ*. Related studies have shown that BPA can increase the transcription of estrogen receptors, leading to precocious puberty [43]. Our findings align with those reported in previous studies. Interestingly, we observed no significant increase in *ERβ* expression in PND 21 mice. However, in PND 45 mice, exposure to BPA at doses of 20 mg/kg or higher significantly elevated *ERβ* expression in the ovaries. This suggests that BPA’s impact on ovarian development varies during lactation, indicating a differential sensitivity to BPA at different developmental stages. Further research is still needed to elucidate this issue.

The comparative analysis of ovarian RNA from the control group (Group A) and the BPA-exposed group (Group G) highlighted distinct gene expression profiles. In the ovaries of 21-day-old mice, 81 significant DEGs were identified in response to BPA exposure: 38 genes were up-regulated, while 43 genes were down-regulated. In contrast, the 45-day-old offspring displayed a different expression pattern with 60 significant DEGs, comprising 36 up-regulated and 24 down-regulated genes. These results suggest that BPA exposure leads to significant alterations in gene expression within the ovaries, with the nature of these changes varying according to the developmental stage of the mice. The result is consistent with some previous studies [44]. KEGG enrichment analysis was conducted to gain insight into the functional consequences of these differential gene expressions. Notably, the analysis revealed significant enrichment of ribosomal metabolic pathways in both 21-day-old and 45-day-old mice ovaries. The ribosomal metabolic pathway is vital in protein synthesis and cell growth [45]. The observed changes in the expression of ribosomal proteins in response to BPA exposure may have implications for ovarian development and function. In 21-day-old mice ovaries, the expression of specific ribosomal proteins, namely L3, L21, and Sa, was significantly down-regulated, while genes encoding ribosomal proteins such as S2, S28, L26, L32, and L10a were up-regulated. In 45-day-old mice ovaries, the expression of ribosomal proteins L21 and Sa was also down-regulated, while genes encoding ribosomal proteins L3, S2, L7a, S26, and L26 were up-regulated. These changes in ribosomal protein expression may significantly impact protein synthesis and, consequently, ovarian development and function.

To thoroughly investigate the complex effects of BPA, this study utilized transcriptome sequencing to compare the ovaries of the 50 mg/kg BPA group with those of the control group. The findings revealed significant alterations in the ribosomal pathway. In both PND 21 and PND 45 mice, genes *Rpl21* and *Rpsa* were notably down-regulated, while Rps2 was up-regulated. Real-time PCR was conducted to assess *Rps2*, *Rpl21*, and *Rpsa* expression levels across all seven groups for both PND 21 and PND 45 mice. The results indicated a dose-dependent decrease in *Rpl21* and *Rpsa* in both age groups, aligning with the transcriptome sequencing findings. However, the impact of BPA on *Rps2* varied with age and dose: in PND 45 mice, *Rps2* expression increased with rising BPA doses. For PND 21 mice, *Rps2* expression was significantly higher than the control at BPA doses ranging from 0.05 mg/kg to 20 mg/kg. At the highest exposure level of 50 mg/kg, *Rps2* expression exceeded that of the control group, indicating a distinctive response based on developmental stage and BPA concentration. The *Rpl21* gene encodes the L21 protein of the ribosomal 60 S subunit, which is involved in embryonic mouse tooth development [46]. Other studies have shown that *Rpl21* is involved in cell proliferation and cell cycle arrest [47]. In the current study, the significant down-regulation of *Rpl21* and *Rpsa* in the ovaries of mice after exposure to BPA may be the cause of BPA affecting ovarian development. *Rps2* encodes the protein S2 of ribosomal 40 S subunit, which is involved in binding aminoacyl transfer RNA to ribosomes to affect the fidelity of mRNA translation, and its increased expression is associated with cell proliferation [48]. The expression trend of *Rps2* in the PND 21 mice ovary confirmed this phenomenon, but the expression of *Rps2* in the PND 45 mice ovary was not the same. Therefore, we speculate that the different manifestations of BPA were related to the dose of exposure and the age of the animals.

In the present study, we confirmed the effect of low-dose exposure to BPA on ovarian development in mice. We confirmed by transcriptome sequencing that ribosome was an essential target for BPA affecting ovarian development. In addition, under the same BPA exposure conditions, although the sera of adolescent mice had higher BPA levels than those of nursing mice, the ovaries of nursing mice showed a higher apoptotic index. This may be because immature mice have a better immune system and resistance than nursing mice to cope with adverse conditions. This study suggests we should classify safe doses of BPA or other environmental estrogens by age, as baby individuals tend to have lower resistance.

## Figures and Tables

**Table 1:** Real-time PCR primer sequences.

Gene ID		Sequence (5′-3′)	GC%	Tm	Length (bp)
*Rps2*	F	CCTGATGATGGCCGGTATAGATG	52.2	64.7	133
	R	TTTCCAGAGGTCGGGAGTCAG	57.1	63.9	
*Rpl21*	F	CCGTGGGCATCATTGTCAAC	55	64.6	71
	R	CTCAATCCGCACATTGATCCTC	50	64	
*Rpsa*	F	ACTTAGGTGGCACCAACCTTGAC	52.2	63.7	117
	R	GAGCTGCGAGCAACAGCTTC	60	63.8	
*β-Actin*	F	TCCTTCCTGGGCATGGAGT	57.9	63	104
	R	AGCACTGTGTTGGCGTACAG	55	60	

Note: was utilized as the internal reference gene for this study.

**Table 2:** Body weight, ovarian weight, and ovarian organ index.

Group	21 Days Old Mice			45 Days Old Mice		
	Body Weight (g)	Bilateral Ovarian Weight (mg)	Ovarian Organ Index (mg/g)	Body Weight (g)	Bilateral Ovarian Weight (mg)	Ovarian Organ Index (mg/g)
Group A	16.67 ± 0.53 ^bc^	11.57 ± 0.58 ^a^	0.70 ± 0.05 ^a^	30.39 ± 2.54 ^a^	36.80 ± 4.31 ^a^	1.20 ± 0.06 ^a^
Group B	19.03 ± 2.21 ^c^	13.11 ± 1.39 ^ab^	0.69 ± 0.01 ^a^	32.94 ± 1.87 ^a^	40.83 ± 2.52 ^a^	1.24 ± 0.07 ^a^
Group C	17.83 ± 1.46 ^b^	15.60 ± 2.97 ^b^	0.87 ± 0.16 ^ab^	31.54 ± 0.19 ^a^	36.57 ± 2.54 ^a^	1.16 ± 0.08 ^a^
Group D	18.40 ± 1.60 ^c^	15.70 ± 1.70 ^b^	0.85 ± 0.06 ^ab^	29.44 ± 2.56 ^a^	34.87 ± 7.29 ^a^	1.17 ± 0.17 ^a^
Group E	14.23 ± 0.67 ^a^	15.67 ± 1.10 ^b^	1.10 ± 0.11 ^b^	32.02 ± 1.45 ^a^	36.90 ± 7.26 ^a^	1.14 ± 0.19 ^a^
Group F	14.47 ± 0.59 ^a^	15.07 ± 2.16 ^b^	1.04 ± 0.11 ^b^	33.15 ± 1.34 ^a^	39.8 ± 4.88 ^a^	1.19 ± 0.11 ^a^
Group G	14.87 ± 0.67 ^a^	16.32 ± 1.26 ^b^	1.10 ± 0.05 ^b^	35.71 ± 1.93 ^a^	45.93 ± 3.26 ^a^	1.29 ± 0.07 ^a^

Note: Group A served as the control group. Group B received 0.05 mg/kg BPA, Group C 0.5 mg/kg, Group D 5 mg/kg, Group E 10 mg/kg, Group F 20 mg/kg, and Group G 50 mg/kg BPA. There were 20 mice in each group. Different lowercase letters indicate significant differences between values in each column.

**Table 3:** Results of apoptosis of offspring ovarian cells induced by BPA.

Groups	Ovary at PND 21 (Gray Value)	Ovary at PND 45 (Gray Value)
Group A	72.61 ± 3.50 ^a^	10.22 ± 1.35 ^a^
Group B	71.22 ± 5.57 ^a^	7.69 ± 2.33 ^a^
Group C	90.00 ± 4.82 ^ab^	12.56 ± 6.77 ^a^
Group D	81.72 ± 7.62 ^a^	20.91 ± 6.97 ^ab^
Group E	77.17 ± 3.28 ^a^	27.14 ± 4.74 ^ab^
Group F	106.13 ± 10.52 ^b^	31.83 ± 12.13 ^ab^
Group G	131.71 ± 6.27 ^c^	38.83 ± 12.25 ^b^

Note: Image J software obtained the optical density value in the table to judge the quantitative degree of apoptosis. The statistical significance analysis was conducted using SPSS 19 software. In the results, different lowercase letters represent significant differences between the groups (*p* < 0.05). Group A served as the control group. Group B received 0.05 mg/kg BPA, Group C 0.5 mg/kg, Group D 5 mg/kg, Group E 10 mg/kg, Group F 20 mg/kg, and Group G 50 mg/kg BPA. There were 20 mice in each group. Different lowercase letters indicate significant differences between values in each column.

**Table 4:** The *ERβ* expressions in the ovary.

Groups	PND 21 *ERβ* (Gray Value)	PND 45 *ERβ* (Gray Value)
Group A	13.05 ± 3.21 ^a^	8.61 ± 0.72 ^a^
Group B	13.37 ± 4.46 ^a^	9.40 ± 2.64 ^a^
Group C	8.81 ± 1.51 ^a^	7.45 ± 1.92 ^a^
Group D	8.25 ± 0.13 ^a^	4.74 ± 1.44 ^a^
Group E	15.90 ± 2.32 ^a^	8.72 ± 1.63 ^a^
Group F	20.02 ± 7.60 ^a^	30.94 ± 14.90 ^b^
Group G	20.67 ± 7.09 ^a^	42.12 ± 8.38 ^b^

Note: Group A served as the control group. Group B received 0.05 mg/kg BPA, Group C 0.5 mg/kg, Group D 5 mg/kg, Group E 10 mg/kg, Group F 20 mg/kg, and Group G 50 mg/kg BPA. There were 20 mice in each group. Different lowercase letters indicate significant differences between values in each column.

## Data Availability

The data for this study are available upon request from the corresponding authors. The RNA-seq data is available in the NCBI dataset (ID: SUB14670619).

## Supplementary Materials

The following supporting information can be downloaded at: https://www.mdpi.com/article/10.3390/toxics12090627/s1, Table S1: F1 reproductive capabilities in different BPA dose groups; Table S2: F2 reproductive capabilities in different BPA dose groups; Table S3: Water intake and weight of female mice during pregnancy; Table S4: Water intake and overall weight of female mice and offspring during lactation.
