# Supplementary material for: Bisphenol A Disrupts Ribosome Function during Ovarian Development of Mice
Source: Toxics. 2024 Aug 26;12(9):627. doi: 10.3390/toxics12090627 (PMC11435667; doi:10.3390/toxics12090627)
Supplement: Supplementary file 1 [file toxics-12-00627-s001.zip › toxics-3157006-supplementary.pdf]

Table S1. F1 reproductive capabilities in different BPA dose groups

Groups (n=20)	Average fetus number	Average fetus weight	Female/male rate
Group A	15.24±0.47[a]	1.98±0.02[a]	1.00
Group B	13.50±1.14[a]	2.01±0.07[a]	0.78
Group C	13.50±0.92[a]	1.91±0.03[a]	0.80
Group D	13.38±1.40[a]	2.01±0.10[a]	0.90
Group E	13.38±1.12[a]	1.96±0.04[a]	0.90
Group F	14.00±0.90[a]	2.02±0.07[a]	0.83
Group G	13.02±0.55[b]	1.91±0.05[a]	0.74

Note: Group A was control group, group B was 0.05 mg • kg^{-1}• d^{-1}, group C was 0.5 mg • $kg\text{-}^{1}$• d^{-1}, group D was 5 mg • kg^{-1}• d^{-1}, and group E was 10 mg • kg^{-1}• d^{-1} Group d, group F was 20 mg • kg^{-1}• d^{-1}, group G was 50 mg • kg^{-1}• d^{-1}. Different lowercase letters indicate a significant difference (P<0.05).

Table S2. F2 reproductive capabilities in different BPA dose groups

Groups	Average fetus number	Death rate %	Female/male rate
Group A (n=20)	14.80±0.13[a]	0	1.00
Group B (n=20)	11.56±0.62[a]	20	1.06
Group C (n=28)	9.10±0.80[b]	40	0.77

Note: Group A was the control group, group B was 0.05 mg • kg-1• d-1, and group C was 50 mg • $kg\text{-}^{1}$• d^{-1}. Different lowercase letters indicate a significant difference (P<0.05).

Table S3. Water intake and weight of female mice during pregnancy

Pregnancy time (n=20)	0 d	3 d	6 d	9 d	12 d	15 d	18 d
Water intake (mL)	6.34±0.69	6.34±0.98	7.12±0.81	7.58±0.77	8.81±0.72	10.4±1.05	11.23±1.70
Body weight (g)	37.72±1.77	37.99±0.96	40.89±0.67	43.74±1.25	51.23±3.95	61.76±5.74	75.66±6.30
Water intake/Body weight	0.17±0.00	0.17±0.01	0.17±0.00	0.17±0.00	0.17±0.00	0.17±0.00	0.15±0.00

Table S4. Water intake and overall weight of female mice and offspring during lactation

Offspring age (n=20)	0 d	3 d	6 d	9 d	12 d	15 d	18 d	21 d
Water intake (mL)	11.70±0.54	20.87±0.94	27.34±1.29	29.36±1.32	32.30±1.32	32.48±1.23	51.04±1.92	63.36±1.76
Whole nest body weight (g)	81.39±1.14	99.91±1.51	123.75±2.31	143.45±2.37	162.91±2.64	176.00±2.98	284.96±9.50	340.07±5.77
Water intake/Whole nest body weight	0.14±0.00	0.21±0.00	0.22±0.00	0.20±0.00	0.20±0.00	0.18±0.00	0.18±0.00	0.19±0.00
